# Platelet function in brown bear (Ursus arctos) compared to man

**DOI:** 10.1186/1477-9560-8-11

**Published:** 2010-06-02

**Authors:** Ole Fröbert, Kjeld Christensen, Åsa Fahlman, Sven Brunberg, Johan Josefsson, Eva Särndahl, Jon E Swenson, Jon M Arnemo

**Affiliations:** 1 Department of Cardiology, Örebro University Hospital, Sweden; 2 Department of Clinical Sciences, Faculty of Veterinary Medicine and Animal Science, Swedish University of Agricultural Sciences, Uppsala, Sweden; 3 Department of Veterinary Clinical and Diagnostic Sciences, Faculty of Veterinary Medicine, University of Calgary, Calgary, Canada; 4 The Scandinavian Brown Bear Research Project, Tackåsen, Orsa, Sweden; 5 Department of Clinical Medicine, School of Health and Medical Sciences, Örebro University, Sweden; 6 Department of Ecology and Natural Resources Management, Norwegian University of Life Sciences, Ǻs, Norway. and Norwegian Institute for Nature Research, Trondheim, Norway; 7 Department of Wildlife, Fish and Environmental Studies, Faculty of Forest Sciences, Swedish University of Agricultural Sciences, Umeå, Sweden; 8 Faculty of Forestry and Wildlife Management, Hedmark University College, Campus Evenstad, Norway

## Abstract

**Background:**

Information on hemostasis and platelet function in brown bear (*Ursus arctos*) is of importance for understanding the physiological, protective changes during hibernation.

**Objective:**

The study objective was to document platelet activity values in brown bears shortly after leaving the den and compare them to platelet function in healthy humans.

**Methods:**

Blood was drawn from immobilized wild brown bears 7-10 days after leaving the den in mid April. Blood samples from healthy human adults before and after clopidogrel and acetylsalicylic acid administration served as control. We analyzed blood samples by standard blood testing and platelet aggregation was quantified after stimulation with various agonists using multiple electrode aggregometry within 3 hours of sampling.

**Results:**

Blood samples were collected from 6 bears (3 females) between 1 and 16 years old and from 10 healthy humans. Results of adenosine diphosphate, aspirin, and thrombin receptor activating peptide tests in bears were all half or less of those in humans. Platelet and white blood cell counts did not differ between species but brown bears had more and smaller red blood cells compared with humans.

**Conclusion:**

Using three different tests, we conclude that platelet function is lower in brown bears compared to humans. Our findings represent the first descriptive study on platelet function in brown bears and may contribute to explain how bears can endure denning without obvious thrombus building. However, the possibility that our findings reflect test-dependent and not true biological variations in platelet reactivity needs further studies.

## Introduction

Hibernating Scandinavian brown bears (Ursus arctos) stay inside their winter dens for approximately 5-7 months [1,2] and during this hibernation period they do not eat, drink, defecate, urinate or have any physical activity. Despite this, brown bears do not develop deterioration in heart function [3], muscle atrophy [4], osteoporosis (black bear, Ursus americanus, observation [5]), or decubitus ulcer (authors observation). Immobility predisposes humans to thromboembolism [6] but in accordance with findings in other hibernating animals [7] it is unlikely that denning bears develop coagulation disturbances. In contrast to most other hibernating animals, the brown bear sustains a body temperature (31-35°C) near normal during hibernation [8-10]. How the brown bear tolerates the physiological extremes related to hibernation is unknown.

Information on the coagulation system in bears is scarce. Previous studies have studied haptoglobin and α_2_-macroglobulin and found both parameters to rise in brown and black bears (Ursus americanus) during hibernation as compared to the active state [8,9,11,12]. We believe that the hibernating brown bear can act as a biological model for insight into the mechanisms of cardiovascular disease in humans. Because physical inactivity and lying flat on the ground are thrombogenic and because the brown bear apparently is free from thromboembolic events we hypothesized that brown bears would demonstrate low platelet activity shortly after leaving the den. In order to characterise the physiological impact we compared our findings to human platelet function.

## Methods

### Material

In mid-April 2009, approximately 7-10 days after leaving the den, wild brown bears were immobilized from a helicopter by darting with a mixture of tiletamine-zolazepam and medetomidine [13]. Blood was drawn from the jugular vein in accordance with previous reports [14]. The study of bears was approved by the Swedish Ethical Committee on animal research

Blood samples from healthy human adult volunteers were collected from the antecubital vein. None of the volunteers were taking any regular medication and none had ingested acetylsalicylic acid for at least a week before blood sampling. Blood was drawn before and 24 hours after an oral loading dose of 600 mg of clopidogrel and, following at least a 10-day interim period, before and 24 hours after an oral loading dose of 600 mg of acetylsalicylic acid. The study done of humans was approved by the regional ethical committee and each subject gave written informed consent.

#### Routine blood cell count

Automated blood count was carried out using flow cytometry (XE-5000, Sysmex Corporation, Kobe, Japan).

#### Platelet aggregometry

Whole blood was drawn into 3 ml plastic syringes containing lepirudin (25 μg/ml, Refludan, hirudin blood collection tubes, Dynabyte, München, Germany) and analyzed by multiple electrode platelet aggregometry between 0.5 and 3 hours after sampling. Electrical aggregometry measures the increase in impedance between a pair of metal electrodes immersed in diluted whole blood. The increase in impedance correlates with the amount of platelets aggregating on the electrodes after adding a platelet agonist to the diluted whole blood. The method correlates well with light transmission aggregometry [15]. Multiple electrode aggregometry was performed at 37°C with a constant stir bar speed of 1000 rpm on a multiple platelet function analyzer (Multiplate impedance aggregometer, Dynabyte). In a polycarbonate cuvette 300 μL of 0.9% NaCl and 300 μL of whole blood were incubated for three minutes before 20 μL of the agonist was added. Aggregation was started by adding adenosine diphosphate (ADP) to obtain a concentration of 6.4 μmol/L, arachidonic acid (ASPI-test) to obtain a concentration of 0.5 mM or thrombin receptor activating peptide (TRAP) to obtain a concentration of 32 μM. Platelet aggregation expressed as increase in impedance (W) was continuously recorded for 6 minutes. Impedance, and thus aggregation is quantified as arbitrary aggregation units (AU) after 6 minutes and the area under the curve of arbitrary units (AUC).

#### Statistics

Values are presented as mean ± standard error of the mean (SEM). The Holm-Sidak one-way analysis of variance (ANOVA) multiple comparisons test was used for pairwise comparisons between bears and humans (baseline, acetylsalicylic acid and clopidogrel). Bivariate associations were examined by least square linear regression. Differences were considered statistically significant when P < 0.05.

## Results

### Haematological parameters

Blood samples were collected from 6 brown bears (3 females) between 1 and 16 years old and from 10 healthy human volunteers (3 females) between 28 and 58 years old. The number of platelets and white blood cells did not differ between species (Additional file 1; Table S1). Compared with humans, brown bears had more and smaller red blood cells with less haemoglobin per cell but with more haemoglobin per volume unit of blood because of the higher number of cells.

### Platelet aggregometry

**Figure 1:**
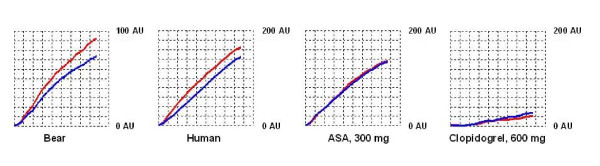
**Traces of platelet function analyzer testing following adenosine diphosphate stimulation in a 4-year old male brown bear (left) and in a 58-year old healthy female volunteer before and after the administration of 300 mg of acetylsalicylic acid (ASA) and 600 mg of clopidogrel**. Platelet aggregation is expressed as increase in impedance after 6 minutes quantified as arbitrary aggregation units (AU). The mean of two runs (red and blue curves) was used. Please note different scaling.

In humans, ADP stimulation (AU or AUC) was unaffected by 300 mg of acetylsalicylic acid. Clopidogrel 600 mg, on the other hand, reduced both these numbers by half compared to baseline and to a level almost equal to the brown bear values (Figure 1, Additional file 2; Table S2).

At baseline, results from stimulation with arachidonic acid (ASPI-test) in humans were more than twice the values found in bears. A dramatic reduction in the ASPI-test was seen in humans after acetylsalicylic acid and a modest reduction was seen after clopidogrel (Additional file 2; Table S2).

In humans, only minor non-statistically significant changes in thrombin receptor activating peptide (TRAP) testing were seen after acetylsalicylic acid and clopidogrel and all values were 4-10 times higher than the brown bear values (Additional file 2; Table S2).

There were no statistically significant correlation between age of the bears and aggregometer testing results. The highest r-value for linear regression was 0.27 (P = 0.60) between ADP aggregation units and age (the other r-values were lower and are not shown).

## Discussion

Our main findings on platelet function in Scandinavian brown bears are that all the functional parameters of platelets we investigated by multiple electrode aggregometry were half or less of those of human values. Platelet count did not differ between species.

Until now, information on brown bear coagulation and platelet function has been limited. Two studies have found that haptoglobin is higher in hibernating than non-hibenating black [11] and brown bears [12]. Another large plasma protein, α_2_-macroglobulin, increases clotting time [8,9] and levels of serum α_2_-macroglobulin were found to be significantly higher during hibernation compared to the active state of brown bears [12] and black bears [16], indicating anticoagulation during hibernation.

Current research on comparative hemostasis among species is increasing but for most animals not routinely used in laboratory research this area remains scarcely covered, primarily because of the lack of species-specific antibodies for immunological assays [17]. Older studies have even suggested that quantitative comparisons between hemostasis in different species are illusory, because any difference found will have qualitative and quantitative aspects [18]. A more recent commentary put forward that the basis of blood coagulation in vertebrates involves tissue factor and fibrinogen developed from a primordial system, with all the other factors "sandwiched" in later during evolution [19]. Because the main goal of aggregation is the binding of fibrinogen to the platelet receptor glycoprotein IIb-IIIa, this hypothesis opens for functional testing of platelet aggregatory responses to agonists in vitro in most species. Everything boils down to formation of the thrombus and, given that relevant agonists are used, between-species comparisons can be made - for example by aggregometer testing. In a comparative aggregometer study comparing human, dog and calf blood, the authors concluded that ADP and collagen are the agonists of choice giving the most consistent results between species [20]. A recent study using aggregometer testing in llamas (*Lama glama*) demonstrated that platelets from this animal were responsive to ADP but unresponsive to arachidonic acid [21].

Our aggregometer findings in bears were consistent; all bears demonstrated lower platelet function values than in humans. Adenosine diphosphate, aspirin and thrombin receptor activating peptide tests were all statistically significantly lower in bears and the test values were comparable to values obtained in humans following platelet inhibition with clopidogrel or acetylsalicylic acid administration in this study and as demonstrated by others [22]. In our small material, there was no apparent systematic difference in platelet function between adult bears and yearlings or between sexes. All our samples were taken in mid April and must be considered as a snapshot of platelet function. Therefore we cannot comment on whether platelet function changes during denning but any possible hibernation effect might still be present in our material, because sampling was conducted so soon after leaving the den.

The physiological significance of our findings could be straightforward; the apparently low platelet aggregation in brown bears might help them to sustain 6 months of winter sleep lying on the ground with limited physical activity obviously without significant thromboembolic events. The platelet number in bear blood (demonstrating values within the human range) and functional testing with three different agonists using platelet aggregometer (with consistently lower values compared with humans) could indicate spontaneous reduced platelet function in brown bears compared to humans. However, the possibility that our findings reflect test-dependent and not true biological variations in platelet reactivity needs further studies.

A potential source of error in our assessment of platelet function in brown bears is the effect of the anaesthetics used. The alpha-2 agonist medetomidine does not alter platelet aggregation [23] while no studies are available for the tiletamine-zolazapam combination on coagulation and platelet activation. However, higher cardiac and stroke indices have been observed in rats with tiletamine-zolazapam than with ketamine and pentobarbital [24]. For xylazine-tiletamine-zolazapam one study on horses found a reduction in platelet count after 10 minutes that returned to normal within 30-60 minutes whereas a xylazine-diazepam-ketamine combination resulted in an increased platelet count after 10 minutes [25]. From these data we find it reasonable to conclude that titelamine-zolazapam in combination with medetomidine most likely had a neutral effect on platelet aggregation

A limitation of our study was that we did not measure specific clotting factors or complementary characterisation of platelet function in order to characterize what specific part of aggregation is inhibited in bears. By performing all sampling at the same time of the year we provided a snapshot of platelet function that is not generalizable to other times of the year - e.g. in autumn following excessive intake of bilberries (Vaccinium myrtillus) and crowberries (Empetrum hermaphoditum) (both might inhibit platelet function [26]) or while the bears lie inactive in the den (theoretically predisposing them to thromboembolism [6]). Also, we did not characterize bear platelet ultrastructural anatomy. Our study was conducted to test biological relevance and feasibility and fieldwork logistics in order to formulate an adequately substantiated application for ethical approval to perform research on hibernating brown bears. Recently this ethical approval was granted to us and we hope in the future to be able to present data on platelet function in the same bears in hibernation and in the active state.

We conclude that platelet function values using ADP, ASPI or TRAP are lower in Scandinavian brown bears than in humans. Our findings represent the first descriptive study on platelet function in brown bears and may reflect true biological variation, but test specific differences cannot be excluded. More studies are needed to clarify whether these findings may contribute to explain how bears can endure denning without obvious thrombus building.

## Competing interests

The authors declare that they have no competing interests.

## Authors' contributions

OF designed the study, participated in bear blood sampling, performed statistical analysis and drafted the manuscript. KC designed the platelet part of the study, participated in human and bear blood sampling, and was responsible for thrombocyte function testing. ÅF participated in bear blood sampling and immobilization and participated in manuscript writing. SB was responsible for all bear handling and planning of the bear part of the study. JJ performed platelet investigations, and participated in human and bear blood sampling. ES participated in study design and -analysis and participated in manuscript drafting. JES was responsible for bear project coordination, participated in analysing the results and in manuscript drafting. JAM was responsible for bear blood sampling and immobilization and participated in manuscript writing. All were involved in manuscript revising for important intellectual content and approved the final version.

## Supplementary Material

Additional file 1 **Table S1**. Hematological parameters. Scandinavian brown bears vs. humans. Click here for file

Additional file 2 **Table S2**. Platelet aggregometry in Scandinavian brown bears and humans. Click here for file
